# Is adiposity associated with objectively measured physical activity and sedentary behaviors in older adults?

**DOI:** 10.1186/s12877-020-01664-y

**Published:** 2020-07-28

**Authors:** Wenfei Zhu, Zhiwei Cheng, Virginia J. Howard, Suzanne E. Judd, Steven N. Blair, Yuliang Sun, Steven P. Hooker

**Affiliations:** 1 School of Physical Education, Shaanxi Normal University, No. 620, West Chang’an Avenue, Chang’an District, Xi’an, 710119 Shaanxi China; 2 Department of Epidemiology, School of Public Health, University of Alabama at Birmingham, Birmingham, AL USA; 3 Department of Biostatistics, School of Public Health, University of Alabama at Birmingham, Birmingham, AL USA; 4 Departments of Exercise Science and Epidemiology/Biostatistics, Arnold School of Public Health, University of South Carolina, Columbia, SC USA; 5 College of Health and Human Services, San Diego State University, San Diego, CA USA

**Keywords:** Adiposity, Physical activity, Stationary time, Accelerometer, Older adults

## Abstract

**Background:**

Many older adults in the U.S. do not achieve the recommended amount of physical activity (PA) to fully realize a myriad of health benefits. Adiposity is one of those important correlates of PA and sedentary behaviors. However, the full extent to which adiposity is associated with PA and stationary time (STA) is uncertain. Therefore, we examined the association of adiposity with objectively measured PA and STA in black and white older adults.

**Methods:**

We conducted a cross-sectional study of older adults enrolled in the REasons for Geographic and Racial Differences in Stroke (REGARDS) Study 2003–2007 who participated in an ancillary accelerometer study 2009–2013. Assessment of body mass index (BMI) and waist circumference (WC) was completed during an in-home visit in the parent study. PA was measured by Actical™ accelerometers, which provided estimates of moderate-to-vigorous-intensity PA (MVPA), light-intensity PA (LPA), and STA for 4–7 consecutive days. Data from accelerometers were standardized to square root percentages of total wear time per day (SqrtMVPA%, SqrtLPA%, and SqrtSTA%). Interactions were tested for BMI and WC by race and sex, separately.

**Results:**

Data were available for 7873 participants (69.8 ± 8.7 yr, 54.2% women, 31.5% African American). In mixed linear regression models, significant interactions existed in BMI by race and sex for the SqrtMVPA%, WC by race and sex for the SqrtMVPA% and the SqrtLPA% model(*p* < 0.05). No interaction was significant for the logistic model of meeting the PA guideline or not. In subgroup analyses, BMI was inversely associated with SqrtMVPA%, SqrtLPA%, and positively related to SqrtSTA% in black women, white men and white women after adjustments. Similar patterns were observed between WC and SqrtMVPA%, SqrtLPA%, and SqrtSTA% in all groups, respectively. However, BMI was not associated with SqrtMVPA% in black men. Those with higher BMI or WC were less likely to meet the PA guideline in all groups.

**Conclusions:**

Adiposity was inversely associated with higher levels of MVPA/LPA and positively associated with higher levels of STA among black and white older adults. Prevention efforts aimed at promoting weight control may be beneficial to prevent physical inactivity and sedentary lifestyle among older adults.

## Background

Epidemiological studies [1–4] have shown that physical activity (PA) is associated with improved physical function, lower prevalence of several chronic diseases, and reduced all-cause premature mortality. The Physical Activity Guidelines for Americans (second edition) [5] recommends adults should do at least 150 min to 300 min a week of moderate-intensity, or 75 min to 150 min a week of vigorous-intensity aerobic activity, or an equivalent combination. The guidelines also emphasize the importance of avoiding prolonged sitting, and provide some new information and guidance on the benefits and practices of moving more and sitting less.

A large proportion of individuals, especially older adults, have very limited PA combined with prolonged stationary time (STA), and thus fail to gain the subsequent health benefits. For example, analyses from the 2003–2004 National Health and Nutritional Examination Survey (NHANES) indicated that the proportion of adherence to the first edition PA guidelines (30 or more minutes of MVPA on 5 of 7 days, accumulated in modified 10-min bouts) was only 2.4% ± 0.4 among U.S. older adults aged ≥60 yrs. [6]. A later study [7] using data from NHANES 2005–2006 reported that the proportion of older adults aged 60–69 meeting the recommended level of PA (150 min of MVPA in total per week, accumulated in modified 10-min bouts) was 8.5% ± 1.5 for accelerometer measures, while the proportion was 6.3% ± 1.5 for those aged ≥70 yrs. Analyses from the REGARDS cohort [8] using acceleometry revealed only 6–22% of the older adults aged ≥45 yrs. accumulated at least 150 min of MVPA per week (accumulated in 1-min bouts to reflect the importance of accumulation in older adults). Women and African Americans were more likely to fail to meet the PA guidelines [6, 7]. Other analyses found a significant portion of deaths [2] and higher percentage of health care expenditures [1] were associated with inadequate PA. Thus, more efforts are needed to support and implement programs, practices, and policies to increase PA.

An increasing body of literature has focused on why some individuals do not engage in sufficient PA. Some studies [9–13] have identified demographic, biological, psychological, social, and environmental variables linked to PA levels. However, most studies were based on self-reported PA, which may provide imprecise estimates and impede the understanding of how to promote PA, especially for the highly sedentary population. Objective assessment of PA using accelerometers can overcome many challenges of measuring PA and offers the potential to explain the variances of PA and STA.

Adiposity may be a significant factor related to PA/STA levels. Cross-sectional and longitudinal studies [14–21] have shown less moderate-to-vigorous-intensity PA (MVPA) and light-intensity PA (LPA) and more STA are associated with higher level of adiposity, usually measured by body mass index (BMI) or waist circumference (WC). Using the method of Dual Energy X-ray Absorptiometry, research [22] also showed PA is inversely associated with body fat and positively associate with fat-free mass. Furthermore, some recent studies suggest the association between PA and adiposity may be bidirectional, and obesity/overweight may actually predict or precede a lower level of PA and more STA. For example, higher BMI was consistently associated with less objectively measured MVPA and more STA in American and Norwegian adults [13, 23]. In a study of children, percent body fat measured by dual energy X-ray absorptiometry was negatively associated with subsequent MVPA level [24]. One study in older population [25] illustrated that obesity was associated with lower levels of physical activity and physical function. However, the extent to how adiposity is associated with objectively measured PA and STA, and the adherence to the PA guidelines in older adults remains uncertain. Moreover, in a national cohort of blacks and white adults aged 45 and older, our research team observed significant differences among race/sex groups for mean STA, LPA, MVPA, and proportion achieving > 150 min/wk. of MVPA [8]. White men had significantly lower STA and higher LPA, MVPA, and proportion achieving > 150 min/wk. of MVPA than other groups. Nevertheless, whether race or sex moderates the relationship between adiposity and PA remains uncertain.

Thus, we investigated the relationships between indicators of adiposity and objectively measured PA and STA, as well as the adherence to the PA guidelines in a large diverse population of black and white older adults. We hypothesized that adiposity would be associated with PA and STA levels, and obesity/overweight would be a contributor to physical inactivity and failure to meet the PA guidelines in older adults.

## Methods

### Study design and sample

Participants in this analyses were enrolled from the REasons for Geographic and Racial Differences in Stroke (REGARDS) Study, a national, population-based study to investigate causes of regional and racial disparities in stroke mortality [26]. From 25 January 2003 to 31 October 2007, black and white adults, aged 45 and older, were recruited by random selection of telephone numbers from a commercially available nationwide list (Genesys, Daly City, CA). Trained interviewers obtained demographic information and medical history through a computer-assisted telephone interview. An in-home brief physical examination was conducted after written informed consent was obtained. Examination included anthropometric measurements, blood pressure, inventory of prescription and nonprescription medications taken within the previous 2 weeks, phlebotomy, and urine collection. All participants gave informed consent, and the study was approved by the institutional review boards of all participating institutions.

### Measures

Age, race, sex, region of current residence (southeastern U.S. Stroke Belt vs. rest of the U.S.), highest education level, smoking (current, never, past), hypertension (systolic blood pressure ≥ 140 mmHg or diastolic blood pressure ≥ 90 mmHg or self-reported use of antihypertensive medications), and diabetes (fasting glucose > 126 mL/dL or nonfasting glucose > 200 mL/dL or self-reported use of oral hypoglycemic medications or insulin) were defined from baseline interview and in-home assessment.

During the in-home visit, using an 8-ft metal tape, height was measured without shoes; wearing light clothes, weight was measured using a standard 300-lb calibrated scale. BMI was calculated as weight (kg) divided by height squared (m^2^) and categorized as normal: under 25 kg/m^2^, overweight: 25 to 30 kg/m^2^, obese: over 30 kg/m^2^. Using a cloth tape over skin or lightweight clothing, waist circumference (WC) was measured at the midpoint between the lowest rib on the right side and the top of the iliac crest. WC was used as an indicator of health risk because abdominal obesity is the primary exposure variable [27].

An ancillary accelerometer study was approved and started in October 2008 for implementation into the ongoing REGARDS study. Detailed design and methods for the study have been described elsewhere [8, 28]. Participants were invited to wear an accelerometer for 7 days for assessment of PA. The protocol used an Actical™ accelerometer (Mini Mitter Respironics, Inc. Bend, OR) worn over the right hip attached to a neoprene waistband. Participants were instructed to put on the device after waking up each morning and take it off prior to going to bed each evening. In the ancillary accelerometer study, 8096 of 12,146 participants who were invited and agreed to wear the accelerometer provided usable data with the criterion of > 4 days with > 10 h/day of wear time [8]. Absolute time spent in sedentary behavior, LPA, MVPA and the proportions of total accelerometer wear time spent in STA (STA%), LPA (LPA%), and MVPA (MVPA%) were determined to standardize accelerometer wear time [8]. The epoch length was set as 60 s (1 min). Time spent in LPA or MVPA was presented for every minute meeting the data processing criterion in order to reflect the importance of accumulation. Activity count cut-points were applied to differentiate STA (0–49 cpm), LPA (50–1064 cpm), and MVPA (> 1065 cpm), respectively [29]. Non-wear periods were defined as a string of zero counts per minute (cpm) for > 120 consecutive minutes. The average time interval between the BMI/WC and PA measures was 5.7 ± 1.5 years. After excluding those missing BMI, WC, or any other covariates of interest (*n* = 222), 7873 remained for final analyses.

### Statistical analyses

Because of the inherent variability in daily accelerometer wear time, MVPA%, LPA%, STA% rather than the absolute time spent in MVPA, LPA and STA were used as the main dependent variables. As MVPA%, LPA%, STA% failed the normality test, we performed square root transformations to ensure normality of distribution and meet the criteria for regression analysis. The square root transformed MVPA% (Sqrt MVPA%), LPA% (Sqrt LPA%), STA% (Sqrt STA%) showed a normal distribution on visual inspection.

Mixed linear regression models were tested for the hypothesized associations with BMI and WC measures as predictors and Sqrt MVPA%, Sqrt LPA%, Sqrt STA% as response variables, with adjustments for age, sex, race, region of residence, education, BMI, hypertension, smoking, diabetes, and time interval between BMI/WC and PA measures, as well as the random effect of intercept and BMI × time intervals between measures. Logistic regression analysis was used to estimate odds ratios (ORs) of not meeting the PA guideline (150 min a week of moderate-intensity, or 75 min a week of vigorous-intensity PA, or an equivalent combination of moderate- and vigorous-intensity PA), and the associated 95% CIs, with adjustment for covariates mentioned above.

We were interested in whether associations differed by race and sex, so we tested interactions with BMI and WC by race and sex subgroups (black men, black women, white men and white women). All probability values were based on 2-tailed tests; *p* < 0.05 indicated statistical significance. Analyses were conducted using SAS version 9.4 (SAS Institute, Cary, NC).

## Results

Characteristics of participants are displayed in Table 1. Among the 7873 participants, 54.2% were women, 31.5% were black, 44.5% were college graduates or above, and 54.5% were from the stroke-belt region of the United States. At baseline of the parent study, 27.7% did not report performing any exercise, 39.8% reported exercising one to three times per week, and 32.4% reported exercising four or more times per week. The mean (±SD) age was 69.8 ± 8.7 years at baseline. Baseline BMI and waist circumference were 28.7 ± 5.7 kg·m^− 2^, and 93.8 ± 14.6 cm, respectively. Participants were compliant wearing the accelerometer for 6.6 ± 0.8 days.

**Table 1:** Characteristics of Participants (*N* = 7873)

Variable	Total
**Age, mean (SD), yr**	69.8 (8.7)
**Women, n (%)**	4267 (54.2)
**Blacks, n (%)**	2483 (31.5)
**Stroke-belt**^**a**^**, n (%)**	4291 (54.5)
**Education, n (%)**	
**Less than high school**	493 (6.3)
**High school graduate**	1757 (22.3)
**Some college**	2119 (26.9)
**College graduate and above**	3504 (44.5)
**Smoking, n (%)**	843 (10.7)
**Hypertension, n (%)**	4066 (51.6)
**Diabetes, n (%)**	1256 (16.0)
Body mass index, mean (SD), kg*m^**−2**^	28.7 (5.7)
**Waist circumference, mean (SD), cm**	93.8 (14.6)
**Systolic blood pressure, mean (SD), mmHg**	125.3 (15.6)
**Diastolic blood pressure, mean (SD), mmHg**	76.1 (9.3)

^a^ denotes a region in the United States comprising the states of North Carolina, South Carolina, Georgia, Mississippi, Alabama, Arkansas, Kentucky, Louisiana, and Tennessee

Table 2 displays the average levels of BMI, WC, and accelerometer-measured variables in different race and sex groups. White women had the lowest BMI, WC, STA, and highest LPA, LPA%, compared to other groups. White men had the highest WC, MVPA and MVPA% among groups. Black men had similar levels of adiposity as white men, and the most total device wearing time, STA and STA%. Black women had the highest BMI, and accumulated the least total device wearing time MVPA, MVPA%, LPA, LPA%, and the most STA%.

**Table 2:** Adiposity, physical activity, and stationary time by race and sex subgroups (Mean (SD))

Variable	Black men (***n*** = 933)	Black women (***n*** = 1550)	White men (***n*** = 2673)	White women (2717)
**Body mass index, kg/m**^**2**^	28.9 (5.1)	31.2 (6.6)	28.1 (4.6)	27.7 (5.8)
**Waist circumference, cm**	98.5 (12.8)	94.3 (14.6)	98.9 (12.1)	86.9 (14.7)
**Total wearing time, min/day**	899.6 (130.1)	884.7 (123.7)	896.2 (94.9)	885.4 (91.3)
**MVPA**^**a**^**, min/day**	12.4 (17.1)	8.1 (12.8)	17.8 (20.8)	12.1 (16.0)
**LPA**^**b**^**, min/day**	181.3 (83.7)	175.6 (80.6)	191.9 (76.8)	194.5 (75.1)
**STA**^**c**^**, min/day**	705.9 (135.0)	701.0 (131.9)	686.5 (110.2)	678.8 (107.6)
**MVPA%**^**d**^	1.4 (1.9)	0.9 (1.4)	2.0 (2.3)	1.3 (1.7)
**LPA%**^**e**^	20.2 (9.0)	20.0 (8.9)	21.5 (8.3)	22.0 (8.3)
**SED%**^**f**^	78.4 (9.9)	79.1 (9.5)	76.6 (9.4)	76.6 (9.1)

^a^ MVPA refers to moderate to vigorous physical activity, indicating minutes in which the accelerometer registered ≥1065 cpm

^b^ LPA refers to light intensity physical activity, indicating minutes in which the accelerometer registered 50–1064 cpm

^c^ STA refers to stationary time, indicating minutes in which the accelerometer registered < 50 cpm

^d^ Denotes the proportion of total accelerometer wear time spent in moderate to vigorous intensity physical activity

^e^ Denotes the proportion of total accelerometer wear time spent in light intensity physical activity

^f^ Denotes the proportion of total accelerometer wear time spent in sedentary behavior

In the mixed linear regression models, significant interactions existed in BMI by race and sex for the Sqrt MVPA%, and WC by race and sex for the Sqrt MVPA% and the Sqrt LPA% (*p* < 0.05). Thus, all results were stratified by race and sex for ease of comparability. In subgroup analyses, BMI was inversely associated with Sqrt MVPA% and Sqrt LPA%, and positively related to Sqrt STA% in black women, white men and white women after adjustments for age, region of residence, education, hypertension, smoking, diabetes, time interval between measures. Similar patterns were observed between WC and Sqrt MVPA%, Sqrt LPA%, and Sqrt STA% in all the groups, respectively. BMI was inversely associated with Sqrt LPA%, and positively related to Sqrt STA% in black men (Table 3). BMI was not significantly associated with Sqrt MVPA% in black men (*p =* 0.792).

**Table 3:** Association^a^ of adiposity with physical activity and stationary time (*N* = 7873)

	Sqrt MVPA%^**b**^			Sqrt LPA%^**c**^			Sqrt STA%^**d**^		
	Beta	P	95% C.I.	Beta	P	95% C.I.	Beta	P	95% C.I.
**Body mass index**									
Black men	0.004	0.792	−0.023, 0.030	− 0.027	**< 0.001**	− 0.038, − 0.015	0.016	**< 0.001**	0.010, 0.023
Black women	− 0.017	**< 0.001**	− 0.021, − 0.013	−0.029	**< 0.001**	− 0.036, − 0.022	0.016	**< 0.001**	0.012, 0.020
White men	−0.036	**< 0.001**	− 0.041, − 0.030	−0.028	**< 0.001**	− 0.035, − 0.021	0.020	**< 0.001**	0.016, 0.024
White women	−0.022	**< 0.001**	− 0.026, − 0.018	−0.021	**< 0.001**	− 0.027, − 0.015	0.015	**< 0.001**	0.012, 0.018
**Waist circumference**									
Black men	−0.009	**< 0.001**	−0.012, − 0.006	−0.012	**< 0.001**	− 0.017, − 0.008	0.008	**< 0.001**	0.005, 0.010
Black women	−0.006	**< 0.001**	− 0.009, − 0.003	−0.012	**< 0.001**	− 0.016, − 0.007	0.006	**< 0.001**	0.004, 0.009
White men	−0.011	**< 0.001**	− 0.015, − 0.008	−0.013	**< 0.001**	− 0.016, − 0.010	0.009	**< 0.001**	0.007, 0.010
White women	−0.007	**< 0.001**	− 0.010, − 0.005	−0.009	**< 0.001**	− 0.011, − 0.006	0.005	**< 0.001**	0.004, 0.007

^a^ Adjusted by age, region of residence, education, hypertension, smoking, diabetes, and periods of between adiposity and physical activity measures

^b^ Denotes the proportion of total accelerometer wear time spent in moderate to vigorous intensity physical activity

^c^ Denotes the proportion of total accelerometer wear time spent in light intensity physical activity

^d^ Denotes the proportion of total accelerometer wear time spent in sedentary behavior

No interaction was significant for the logistic model of meeting or not meeting the PA guideline, but all results were stratified by race and sex for ease of comparability. A significant relationship also existed between BMI and WC and odds of not meeting the PA guideline in black men, black women, white men, and white women (Table 4). Time spent in vigorous PA was very limited in this cohort (mean ± SD for vigorous PA: 0.2 ± 1.7 min/d), indicating those who met the PA guideline were mainly older adults who accumulated ≥150 min of MVPA per week. Participants with higher BMI were less likely to meet the PA guideline (5% less likely in black men, 9% in black women, 11% in white men, and 9% in white women for those with higher BMI, respectively). The same pattern was found for WC in this study (3% less likely in black men, 4% in black women, 4% in white men, and 3% in white women for those with higher WC, respectively).

**Table 4:** Association^a^ of adiposity with odds of not meeting the U.S. physical activity guideline^b^ (*N* = 7873)

	Not meeting the physical activity guideline^**b**^		
	O.R.	95% C.I.	P
**Body mass index**			
Black men	1.05	1.01, 1.09	**< 0.001**
Black women	1.09	1.06, 1.13	**< 0.001**
White men	1.11	1.09, 1.14	**< 0.001**
White women	1.09	1.07, 1.12	**< 0.001**
**Waist circumference**			
Black men	1.03	1.01, 1.05	**< 0.001**
Black women	1.04	1.02, 1.05	**< 0.001**
White men	1.04	1.03, 1.05	**< 0.001**
White women	1.03	1.03, 1.04	**< 0.001**

^a^ Adjusted by age, region of residence, education, hypertension, smoking, diabetes, and periods of between adiposity and physical activity measures

^b^ Accumulated ≥150 min a week of moderate-intensity, or 75 min a week of vigorous-intensity aerobic activity, or an equivalent combination

## Discussion

This study employed objective measurements of BMI, WC, PA, and STA in a U.S. national cohort of 7873 older adults enrolled in the REGARDS study. This is one of the first studies to report an association between adiposity and PA and STA in a population of black and white older adults. Lower adiposity was significantly related to higher levels of accelerometer-measured MVPA and LPA and lower levels of STA. Significant interactions were found among BMI/WC by race, sex in nearly all of the regression models. With the magnitude of associations between BMI and PA/SED generally similar across different race and sex subgroups, except with one difference in black men pertaining to Sqrt MVPA%. Not surprisingly, participants in this study spent most of their accelerometer wear time in STA and LPA, while their time spent undertaking MVPA was extremely limited. A significant relationship also existed between adiposity and odds of meeting the PA guideline. Therefore, prevention efforts aimed at promoting weight control may be beneficial to prevent physical inactivity and sedentary lifestyle among older adults.

The relationship between adiposity and PA has been reported in a number of previous cross-sectional studies [30, 31]. Research has consistently identified a negative relationship between adiposity and levels of PA. While most previous studies assessed self-reported PA, some recent studies with objective measures allowed for more reliable estimates of duration and intensity of PA. For example, one study [15] in the NHANES cohort indicated both self-reported and objectively measured MVPA were independently associated with physiological and anthropometric biomarkers, and objectively measured MVPA showed a stronger association with BMI and WC than self-reported MVPA. The British Regional Heart Study [14] using accelerometer measures reported that reduced MVPA and LPA and increased STA were associated with obesity (waist circumference of > 102 cm), and low muscle mass in older men. It was also reported that greater time spent in light intensity activity and lower STA were associated with lower BMI in the Lifestyle Interventions and Independence for Elders Study [16]. Additionally, data from the cohort of Health Survey for England [19] indicated total self-reported leisure-time sedentary behaviors and TV time were associated with BMI and WC, while accelerometer-measured sedentary behavior was associated with WC in the cohort of Health Survey for England. Analyses in the NHANES cohort [13, 17] have indicated that adults with high BMI had significantly lower objectively measured MVPA than those with intermediate and ideal BMI.

A few previous longitudinal studies also demonstrated that obesity/overweight was a significant predictor of PA level later in life. For instance, results from the Iowa Bone Development Study [24] revealed percent body fat measured by dual energy X-ray absorptiometry was associated with subsequent MVPA in childhood. Large cohort studies among adults in U.K. [32] and Australia [33] also showed that obesity at baseline and weight gain led to future physical inactivity and sedentary behaviors. Intriguingly, an analysis from the Medical Research Council Ely Study [34] reported BMI, WC, and fat mass predicted STA, whereas STA did not predict future obesity at 5.6 years of follow-up in U.K. Another study [23] using accelerometers concluded a high baseline body weight and BMI may determine lower levels of MVPA, but STA and PA did not predict weight gain over 6-year follow-up in Norwegian adults.

BMI and WC are both commonly measured as indicators of adiposity. BMI is highly correlated with directly measured fat mass in older adults, while WC provides information on the degree of visceral obesity. In our study, patterns of association of BMI or WC with PA were similar, except for black men. One possible bias of BMI was that it does not distinguish fat mass and fat-free mass. Research has shown that BMI has limited accuracy to estimate body fat percentage, and it is influenced by sex, age and race/ethnicity [35, 36]. Further investigation is needed to better understand the association between adiposity and PA/STA using objective measures like body fat percentage.

The mechanism of adiposity associated with PA is still uncertain. One possible explanation suggested by Godin et al. [37] is that adiposity may impact PA levels by influencing cognition such as intention/motivation and perceived behavioral control, which is based on the Theory of Planned Behavior. Obese or overweight older adults may be less likely to participate in PA because of low self-efficacy, poor body image, less social support, low proficiency in sports, discomfort from heaviness, and low physical functioning [25, 38]. Once excess fat is accumulated in earlier life, possibly due to a low level of PA, it may lead to even lower PA participation. The association between adiposity and PA has also been supported by a Mendelian randomization analysis [38] and data from animal models [39]. However, we were not able to examine any further mediating factors, such as self-efficacy, social support, or proficiency in sports, in the association between adiposity and PA. Future studies will be necessary to uncover the mechanism of how adiposity may impact later PA behaviors in older adults.

This study has several strengths. First, the sample was recruited from a well-characterized cohort of midlife and older black and white adults living in the U.S. The participants were also extremely compliant with the 7-day protocol (average wear time per week: 6.6 ± 0.8 d), providing a large pool of quality accelerometer-derived data. Second, we used actual measurements to access the adiposity of participants during an in-home visit, providing more detailed analysis for older white and black women and men. Third, an objective measure of PA was applied to examine relationships between adiposity and objectively measured PA and STA among community-dwelling older adults. This allowed for full analyses of time spent in PA of varying intensity. Finally, this is one of the first studies to examine the relationship between adiposity and meeting the PA guideline in older adults.

The findings of this study were also subject to limitations. First, the cohort was quite sedentary, and there was a significant skewness of the variables of PA and STA. The range of MVPA was extremely narrow, so transformations were performed to ensure normality of distribution. Second, there are limitations to using accelerometers, including not being able to identify types and domains of PA or capture upper-body or non-ambulatory movement, potentially resulting in an under-estimation of total PA [40]. Accelerometers calibrated and validated on structured activities performed in a laboratory rather than on free-living activities in our study [41] may underestimate MVPA. Accelerometer counts from a hip worn device can be similar for sitting and standing with negligible movement leading to overestimation of time in sedentary behavior and underestimation of LPA. Third, our results were not generalizable for participants who did not wear the accelerometer for a sufficient number of days/hours or did not agree to wear accelerometer. Some of the participant characteristics collected several years prior to being asked to wear accelerometer may have changed. However, with our large sample size, the changes may not significantly impact the group means, proportions, or association. Finally, as a cross-sectional study, causality cannot be inferred. Future research is needed to explicitly address these issues.

## Conclusion

There was a significant association between adiposity and subsequent PA and STA in this cohort with both black and white older adults. A lower degree of adiposity was significantly related to higher levels of MVPA and LPA, and less STA. BMI and WC were also inversely associated with the odds of not accumulating adequate PA to meet the PA guidelines. Race and sex did not influence in the association between adiposity, and PA/SED. The study suggests tailored PA interventions should be developed among overweight and obese older adults.

## Data Availability

This study uses data from the Reasons for Geographic and Racial Differences in Stroke (REGARDS) cohort. In order to abide by its obligations with NIH/NINDS and the Institutional Review Board of the University of Alabama at Birmingham, REGARDS facilitates data sharing through formal data use agreements. Any investigator is welcome to access the REGARDS data through this process. Requests for data access may be sent to regardsadmin@uab.edu.

## Abbreviations

BMI: Body mass index
CPM: Counts per minute
LPA: Light-intensity physical activity
MVPA: Moderate-to-vigorous-intensity physical activity
ORs: Odds ratios
PA: Physical activity
REGARDS: REasons for Geographic and Racial Differences in Stroke
STA: Stationary time
WC: Waist circumference
